# Effect of particle size, moisture content, and supplements on selective pretreatment of cotton stalks by *Daedalea flavida* and enzymatic saccharification

**DOI:** 10.1007/s13205-016-0548-x

**Published:** 2016-11-03

**Authors:** Harmanpreet Meehnian, Asim K. Jana, Mithu Maiti Jana

**Affiliations:** 1 Department of Biotechnology, Dr B R A National Institute of Technology, Jalandhar, Punjab 144011 India; 2 Department of Chemistry, Dr B R A National Institute of Technology, Jalandhar, Punjab 144011 India

**Keywords:** Cotton stalks, Particle size, Moisture content, Supplements, Delignification, Enzymatic saccharification

## Abstract

A large amount of cotton stalk waste biomass with high cellulose content are incinerated by the farmers causing air pollution. The high cellulose content of cotton stalks can be converted to fermentable sugars by fungal delignification pretreatment of lignocellulosic biomass and enzymatic saccharification. The effect of particle size, moisture content, and media supplements was studied for delignification of cotton stalks by *Daedalea flavida* MTCC 145 (DF-2) in solid-state fermentation. The highest lignolytic enzyme activities, optimal lignin degradation 29.88 ± 0.97% (w/w) with cellulose loss 11.70 ± 1.30% (w/w), were observed in cotton stalks at particle size 5 mm with 75% moisture content after 20 days. Cellulolytic enzyme activity increased with decrease in particle size and increased moisture content. The addition of Cu^2+^, gallic acid, and veratryl alcohol enhanced the lignolytic enzyme production and the lignin degradation. In addition to increased laccase activity, Cu^2+^ inhibited the cellulolytic activity. Supplements Cu^2+^ at 0.5 mM/g gave the best results of lignin degradation 33.74 ± 1.17% (w/w) and highest selectivity value (SV) 3.15 after pretreatment. The glucose yield increased to 127.44 ± 4.56 mg/g from 20 day pretreated cotton stalks with Cu^2+^ supplements, ~threefolds higher than untreated cotton stalks. The study is important for the production of fermentable sugars from cotton stalks residues which can further be utilized in production of bioethanol and other applications.

**Electronic supplementary material:**

The online version of this article (doi:10.1007/s13205-016-0548-x) contains supplementary material, which is available to authorized users.

## Introduction

The expeditious consumption of non-renewable energy resources has awakened the world to harvest the energy from renewable resources for sustainable growth and development (Tiwari et al. 2013). The bioethanol produced from the food crops, such as corn, has been considered as threat for food security; therefore, replacement of it with economical and most abundantly available lignocellulosic biomass mainly agriculture residues is of great interest (Sun and Cheng 2002). The protective shielding of lignin in lignocellulosic biomass restricts the enzymatic saccharification of holo-cellulose (cellulose and hemicellulose) to fermentable sugars (Fermor 1993). A pretreatment process is necessary to remove the lignin and reduce the cellulose crystallinity from lignocellulosic biomass. Various pretreatment methods, such as physical, chemical and biological methods, are used for delignification of biomass to improve the enzymatic saccharification (Zhang et al. 2007). Biological methods using white-rot fungi (WRF) for pretreatment of lignocellulosic biomass have potential to mineralize lignin to CO_2_ and H_2_O (Boyle et al. 1992). This unique property of WRF is due to the expression of lignolytic enzyme system which includes phenol oxidase (laccase), lignin peroxidase (LiP), and manganese peroxidase (MnP).

The delignification of lignocellulosic biomass by WRF is associated with the excessive cellulose loss. During pretreatment of lignocellulosic biomass, WRF produces cellulolytic enzymes and digest the cellulose for its own growth and metabolism resulting low selectivity value (SV, the ratio of lignin degradation to cellulose loss) (Eriksson et al. 1990). The cellulose loss during pretreatment of lignocellulosic biomass by WRF is the major problem, which results in low carbohydrate recovery and low glucose yield after enzymatic saccharification. Pretreatment of cotton stalks with non-selective WRF *Phanerochaete chrysosporium* was studied by Shi et al. 2009. Although high lignin degradation was obtained, but high cellulose loss was also observed resulting low SV and low glucose yield after enzymatic saccharification. High SV is an important parameter for selective lignin degradation and higher enzymatic saccharification (Wan and Li 2010). Various WRF, such as *Daedalea flavida*, *Cereporiopsis subvermispora*, *Phlebia radiata*, *Pleurotus ostreatus* etc., have been reported for high SV due to their intrinsic property (Sharma and Arora 2014; Thakur et al. 2013; Wan and Li 2010). The SV was reported in relation to screening of selective lignin degrading fungal strains (Zhang et al. 2007). The SV of fungal strains is influenced by the production of lignocellulolytic enzymes which varies with the variation in particle size, moisture content, etc. Effect of various media supplements (Cu^2+^, Mn^2+^, ferulic acid, xylidine, veratric acid, vanillic acid, cinnamic acid, guaiacol, etc.) on production of lignolytic enzymes in solid-state fermentation (SSF) has been studied earlier (Liu et al. 2013). Inhibition of cellulolytic enzyme activities by heavy metals, such as Cu^2+^ and Mn^2+^, has been reported earlier (Geiger et al. 1998; Tejirian and Xu 2010). The effect of the above parameters on the production of lignocellulolytic enzymes in relation with SV in pretreatment has not been studied until date.

India is the second largest producer of cotton after China in the world and cultivates the crop in area of 12.19 million hectare (Approx.), which produces abundant lignocellulosic cotton stalk residues as waste. Cotton stalks consisting of 49% holo-cellulose on dry weight basis is good source for production of fermentable sugars (Shi et al. 2012). The present study on pretreatment of cotton stalks by *Daedalea flavida* MTCC 145 is targeted towards achievement of higher delignification of cotton stalks with minimum cellulose loss, i.e., enhanced SV/recovery of carbohydrates using optimal particle size, moisture content, and supplements.

## Materials and methods

### Materials

Fungal culture media (yeast extract glucose agar and potato dextrose agar), birch wood xylan, carboxymethyl cellulose (CMC), *p*-nitrophenyl–β-D-glucopyranoside, and standard sugars (HPLC-grade) were purchased from HiMedia laboratories Ltd, Mumbai, India. Veratryl alcohol was purchased from Sigma Aldrich Co, USA. 2, 2-azino-bis-3-ethylbenzothiazoline-6-sulphonic acid (ABTS) was purchased from MP Biomedicals, USA. All other laboratory reagents were purchased from SD-Fine chemicals Ltd, Mumbai, India.

### Fungal strains

The fungal strains were selected on the basis of their high lignolytic activity and lignin degradation ability reported in the literature (Arora et al. 2002; Shi et al. 2012). WRF strains *Daedalea flavida* NCIM 1087 (DF-1) and *Phanerochaete chrysosporium* NCIM 1106 (PC) were procured from National Collection of Industrial Microorganisms (NCIM), Pune, India. *Daedalea flavida* MTCC 145 (DF-2) and *Trametes hirsuta* MTCC 136 (TH) were procured from microbial-type culture collection (MTCC), Institute of Microbial Technology, Chandigarh, India. DF-1 and PC were grown and maintained in potato dextrose agar medium (potato infusion 20%, dextrose 2%, and agar 1.5% w/v) in petri plates at pH 6, 28 °C. DF-2 and TH were grown and maintained in yeast extract glucose agar medium (yeast extracts 0.5%, glucose 1%, and agar 1.5% w/v) in petri plates at pH 5.8, 25 °C. Strains were sub-cultured after regular period of 15 days.

### Lignocellulolytic ability

The lignolytic ability (ability to degrade lignin by the action of lignolytic enzymes), laccase, and peroxidase activity of WRF were tested using tannic acid (0.1% w/v) (Ander and Eriksson 1977), guaiacol (50 mM) (Hankin and Anagnostakis 1975), and pyrogallol (1% w/v) along with hydrogen peroxide (0.4% v/v) (Egger 1986), respectively, in petri plate cultures. Fungal cultures were grown in their respective agar medium, wells of diameter 8 mm were bored using sterile cork borer in full grown petri plate cultures, and bottom of the wells was sealed with molten agar. Tannic acid 0.5 mL (0.1% w/v), guaiacol 0.5 mL (50 mM), and pyrogallol 0.2 mL (1% w/v) with hydrogen peroxide 0.2 mL (0.4% v/v) were added into the wells. Petri plates were incubated in dark at 28 °C, 15 h for formation of characteristic colored zones of brown, dark red to purple, and golden yellow to brown around the wells because of lignolytic ability, laccase, and peroxidase activity, respectively. The diameters (in mm) of colored zones around the wells were measured as the lignolytic ability, laccase, and peroxidase activity of fungal strains.

Congo red dye test was performed to test the cellulolytic ability of WRF using CMC (10.0 g/L) as sole carbon source (Teather and Wood 1982). The culture plates were flooded with Congo red dye solution (1 mg/L), dye was drained off after 15 min, and the plates were washed three times with NaCl solution (1.0 M). The diameters (in mm) of yellow/halo zones formed along the fungal growth were measured as the cellulolytic ability of fungal strains.

### Solid-state fermentation (SSF) and determination of selectivity value

Cotton (*Gossypium hirsutum*) stalks were collected from the Bathinda region of Punjab, India. Cotton stalks were dried at 40 °C (4% moisture content) in oven, chopped, screened to particle size 1–10 mm, and stored in air-tight containers. The inocula for pretreatment of cotton stalks by SSF were prepared in the liquid culture. Strains were grown by inoculating five culture disks (1 cm diameter each) from the plate into 50 mL liquid medium in 250 mL Erlenmeyer’s flask, incubated (Innova 42R, New Brunswick, USA), and grown stationary at 28 °C, 5 days. Fungal strains were blended aseptically using blender (2000 rpm, 20 s, three times), diluted with sterile medium to obtain dry cell weight (DCW) 1.0 mg/mL, and used as inocula. The fungal biomass DCW concentration in liquid culture was calculated after separation of biomass and taking dry weight. Fungal biomass was separated from liquid culture by centrifugation (4018R, Eppendroff Ltd, USA) at 5000 rpm, 28 °C for 15 min, washed three times with double distilled water, and dried in oven at 40 °C until the constant weight.

Five gram cotton stalks (dried, chopped, and screened to 1–10 mm) were taken in 250 mL Erlenmeyer’s flasks and 15 mL distilled water that were added to maintain 75% moisture content in biomass. Flasks were cotton plugged and autoclaved at 121 °C and 15 psi for 30 min. Flasks containing autoclaved cotton stalks were inoculated with 1 mL inocula (1 mg/mL fungal DCW) equivalent to 0.2 mg fungal DCW per gram cotton stalks and incubated at 28 °C in static condition for 40 days. Samples were withdrawn at 5 day intervals, and SV was calculated by taking ratio of lignin degradation to the cellulose loss. All tests were done in triplicate. Fungal strain having high lignolytic enzyme activities and high SV was selected to study the effect of particle size, moisture content, supplementation on delignification, and enzymatic hydrolysis of cotton stalks.

### Cotton stalks preparation

Cotton stalks were dried, chopped, screened to particle sizes of 1, 5, and 10 mm and stored in air-tight containers for its use in study of the effect of particle size on lignocellulolytic enzyme production, lignin degradation, SV, and enzymatic hydrolysis. The cellulose, hemicellulose, and lignin content of cotton stalks were found to be 37.68, 12.45, and 30.16% (w/w), respectively.

### Pretreatment in solid-state fermentation

Five gram samples of ground cotton stalks for each of the three different particle sizes (1, 5, and 10 mm) were taken in a 250 mL Erlenmeyer’s flask, and different volumes of sodium acetate buffer (20 mM, pH 4.5) were added to maintain moisture content (%w/w) 45, 65, 75, and 85. All flasks were cotton plugged and autoclaved at 121 °C and 15 psi for 30 min. One mL each inocula was added to the flasks. The flasks were incubated at 28 °C in incubator shaker (New Brunswick-INNOVA 42), and samples were withdrawn after the regular interval of 5 days for biomass composition analysis, enzymatic assays, and enzymatic hydrolysis study. To study the effect of supplements on cotton stalks degradation, different concentrations (0.1, 0.5, 1, and 2 mM) of CuSO_4_·7H_2_O, MnSO_4_·5H_2_O, gallic acid, and veratryl alcohol per gram of cotton stalks were supplemented in pretreatment flasks.

### Estimation of enzyme activities

After pretreatment by SSF, cotton stalks with fungal biomass growth in 250 mL flask were suspended in 50 mL of sodium acetate buffer (20 mM, pH 4.5) and extracellular enzymes were extracted using shaker-incubator at 150 rpm and 28 °C for 4 h. The enzyme extracts were filtered using vacuum filtration assembly fitted with nylon membrane (pore size 0.22 µm) and stored at −20 °C.

Laccase activity was estimated using 2, 2-azino-bis-3-ethylbenzothiazoline-6-sulphonic acid (ABTS) as substrate (Bourbonnais and Paice 1990). Lignin peroxidase activity was estimated by oxidation of veratryl alcohol to verataldehyde in the presence of H_2_O_2_ (Kirk et al. 1986). MnP activity was estimated by the oxidation of phenol red in the presence of H_2_O_2_ (Glenn and Gold 1983). One unit of laccase, LiP, and MnP enzyme activities were defined as the amount of enzyme required to oxidize 1 μmol of substrate per min. Carboxymethyl cellulase activity (CMCase/endo-β-1-4-glucanase), filter paper activity (FPase), and xylanase activities were estimated based on dinitrosalicylic acid (DNSA) method (Miller 1959). CMCase and FPase activities were estimated using CMC and Whatman’s no. 1 filter paper as substrate, respectively (Ghose 1987). Xylanase activity was estimated using birchwood xylan as substrate (Bailey et al. 1992). One unit of CMCase, FPase, and xylanase activity was defined as the amount of enzyme required to produce 1 μmol of reducing sugar per min. β-glucosidase activity was estimated using *p*-nitrophenyl- β-D glucopyranoside as substrate and change in absorbance at 405 nm (Parry et al. 2001). One unit of β-glucosidase activity was defined as the amount of enzyme required to release 1 µmol of paranitrophenol (*p*NP) per min. Activities of the enzymes in extract were estimated as IU/mL, and total units of enzymes in the flask were calculated by multiplying the enzyme activity with the volume of buffer added. The activities of the enzyme during pretreatment by SSF were expressed as IU/g solid of untreated cotton stalks.

### Compositional analysis of cotton stalks biomass

Structural carbohydrates (cellulose and hemicellulose) and lignin content in untreated and pretreated cotton stalks were analyzed by the method described in NREL/TP-510-42618 (Sluiter et al. 2011) with some modifications. 0.30 g of biomass samples each was subjected to two-step acid hydrolysis. In the first step, 72% sulphuric acid at 30 °C for 1 h was used. Sample was diluted immediately to 4% of sulphuric acid and autoclaved for 1 h. The solution was filtered using vacuum filtration system fitted with nylon membrane (pore 0.2 µm). The resulting solid residue was washed three times with distilled water, dried (50 °C) to constant weight, and reported as acid-insoluble lignin. The hydrolysis liquor was neutralized by the addition of calcium carbonate and filtered, and sugars in aqueous phase were analyzed by HPLC (Agilent 1200 series, USA) equipped with Agilent Hi-Plex H column and refractive index detector (RID). Sulphuric acid (5 mM) at 0.6 mL/min was used as mobile phase. The temperatures of column and RID detector were maintained at 60 and 55 °C, respectively. Cellulose content was estimated from the glucose concentration using correction factor of 0.9, while hemicellulose content was calculated from xylose, arabinose, mannose, and galactose concentration using correction factor 0.88 for xylose and arabinose and 0.9 for mannose and galactose. Acid soluble lignin was determined by taking absorbance of acid hydrolysis liquor at 205 nm (ε = 110 L/g/cm) using UV–Vis spectrophotometer (Kinetic, Eppendorf Ltd). Ash content of cotton stalk biomass was determined by burning 500 mg of sample in muffle furnace (MFHT-28DXSA, Macro scientific works pvt. Ltd, India) at 575 °C and 3 h to ash, as described in method NREL/TP-510-42622 (Sluiter et al. 2008).

### Enzymatic hydrolysis

Hydrolysis of pretreated and untreated cotton stalks was carried out using commercial cellulase enzymes SaccariSEB EG and SaccariSEB BG obtained as gift from advanced enzymes, Mumbai, India. The endoglucanase activity (using CMC as substrate) of enzyme saccariSEB EG and β-glucosidase activity (using *p*-nitrophenyl-β-D-glucopyranoside as substrate) of enzyme SacchariSEB BG were estimated to be 27.5 and 21.8 IU/mL, respectively. Enzymatic hydrolysis of cotton stalks was carried out by method NREL/TP-5100-63351 (Resch et al. 2015) with some modifications. Enzymes SaccariSEB EG 14 IU/g solid and SaccariSEB BG 11 IU/g solid were loaded to 100 mg of cotton stalks biomass in 10 mL citrate phosphate buffer (50 mM, pH 4.8) in 50 mL falcon tube. 350 μL sodium azide (5% w/v) was added to prevent the microbial growth. The reaction mixture in falcon tubes was incubated in incubator shaker at 150 rpm and 50 °C for 120 h. 1 mL reaction samples were taken at 6, 12, 24, 48, 72, 96, and 120 h, and reactions were stopped by heating the sample vials in water bath at 100 °C and 15 min. The samples were filtered by syringe membrane filters (0.2 µm), and glucose concentrations were estimated by HPLC. The glucose yields were calculated as the milligrams of glucose released per gram of the cotton stalk biomass.

### Characterization of cotton stalks

The morphology of cotton stalks was examined by Scanning Electron Microscope (SEM) (JSM-6510LV, JEOL, Japan) after sputter coating the dried samples with thin film of gold at accelerating voltage 15 kV and magnification 1000×. The crystallinity of cellulose was examined by X-ray diffractometer (X’PERT PRO MPD, Panalytical, Netherland) equipped with sealed tube having Cu Kα radiation source (*λ* = 0.15406 nm) and fixed divergent slit size of 1°. Samples were scanned on a rotating stage between 5° to 50° 2*θ* at step size 0.001° 2*θ* and time per step 20 s. The percentage crystallinity (% Cr) and crystallinity index (C.I.) were calculated from the relations (Segal et al. 1959; Kaith et al. 2009):

$$ {\text{\%  Cr}}\; = \;\varvec{I}_{22} /(\varvec{I}_{22} + \varvec{I}_{18} )\; \times \;100 $$

$${\text{C}} . {\text{I}}.\; = \;\left( {I_{22} \; - \;I_{18} } \right)/I_{22}$$

where *I*_22_ was the intensity of crystalline peak of cellulose at 2*θ* = 22° and *I*_18_ was intensity of amorphous cellulose in cotton stalks at 2*θ* = 18°.

## Results and discussion

### Screening of selective lignin degrading fungal strain

The fungal strains were screened on the basis of their lignocellulolytic abilities and SV in SSF during pretreatment. Diameter of colored zones in tannic acid test, guaiacol test, and pyrogallol test showed that *D. flavida* MTCC 145 (DF-2) was having high lignolytic ability, high laccase, and high LiP activity, respectively (Online Resource 1; Online Resource 2). Congo red dye test showed that DF-2 was having low cellulolytic ability. The SV of DF-2 was higher compared to other fungal strains (Online Resource 3). DF-2 was selected among fungal strains to study the effect of particle size, moisture content, supplements on lignocellulolytic enzyme production, and selective lignin degradation during pretreatment of cotton stalks.

### Degradation of cotton stalks

The effect of particle size, moisture content, and supplements on degradation of cotton stalks biomass during pretreatment was studied.

#### Effect of particle size and moisture content

The effect of particle size and moisture content on degradation of cotton stalks was studied with *D. flavida* MTCC 145 (DF-2). The highest lignin degradation 32.43 ± 3.14% (w/w) with cellulose loss 15.88 ± 4.00% (w/w) was observed in 1 mm cotton stalk particles at 85% moisture content. Lignin degradation 30.09 ± 1.47% (w/w) with cellulose loss 12.75 ± 0.92% (w/w) was observed with particle size 5 mm and moisture content 85% after 20 days (Table 1). Results showed that difference in lignin degradation by reducing the particle size was not significant but cellulose loss increased. There was decrease in the lignin degradation when particle size increased to 10 mm. Cotton stalks having particle size 5 mm were best for optimum lignin degradation with minimum cellulose loss. Small particle size of lignocellulosic biomass inhibits the aeration due to decrease in inter-particle distance which hampers the growth and metabolism of fungi, whereas large particle size of lignocellulosic cotton stalks inhibits the accessibility to nutrients for fungi (Wan and Li 2010). It was observed that DF-2 grew very slowly in cotton stalks at moisture content 45%, degraded less than 3.75% (w/w) lignin and lignin degradation increased with increase in moisture content (Table 1). At higher moisture content, the inter-particle space decreases which hinder the diffusivity of air through lignocellulosic biomass, inhibiting the fungal growth resulting low lignin degradation (Singhania et al. 2009). Cellulose and hemicellulose degradation increased in cotton stalks at higher moisture content and small particle size. The reduction in particle size increased the surface area of cotton stalks exposing accessible holo-cellulose, which was better hydrolyzed at high moisture content and consumed by fungi.

**Table 1:** Effect of particle size and moisture content on degradation of cotton stalks by *D. flavida* (DF-2) (20 days, 28 °C)

PS^a^ (mm)	MC^b^ (%)	Degradation (%)^c^				SV^d^
		Dry mass	Cellulose	Hemicellulose	Lignin	
1	45	5.47 ± 1.01	6.25 ± 0.75	5.68 ± 1.67	3.33 ± 1.53	0.53
	65	14.13 ± 2.53	12.66 ± 1.79	25.21 ± 3.41	24.78 ± 0.91	1.95
	75	15.13 ± 3.28	14.13 ± 2.74	32.93 ± 2.92	30.47 ± 3.31	2.15
	85	14.13 ± 1.10	15.88 ± 4.00	30.08 ± 1.31	32.43 ± 3.14	2.04
5	45	5.80 ± 1.05	5.66 ± 0.49	5.69 ± 0.99	3.43 ± 1.65	0.60
	65	13.53 ± 2.57	11.59 ± 2.08	24.69 ± 3.43	25.19 ± 2.01	2.17
	75	14.60 ± 1.00	11.70 ± 1.30	22.92 ± 1.65	29.88 ± 0.97	2.55
	85	15.53 ± 1.51	12.75 ± 0.92	24.18 ± 1.79	30.09 ± 1.47	2.36
10	45	4.60 ± 0.72	5.26 ± 0.59	4.10 ± 1.51	3.75 ± 1.23	0.71
	65	9.20 ± 2.27	9.89 ± 0.49	20.92 ± 3.20	19.33 ± 2.47	1.95
	75	10.33 ± 2.57	9.91 ± 1.09	18.93 ± 1.05	21.16 ± 0.12	2.13
	85	12.86 ± 1.01	12.19 ± 0.56	21.67 ± 3.01	24.90 ± 3.33	2.04

^a^Particle size

^b^Moisture content

^c^Data are mean values ± standard error of three replicates

^d^Selectivity value

Particle size and moisture content also affected the SV of fungi. The highest SV 2.55 after 20 days of pretreatment was observed in 5 mm cotton stalk particles at 75% moisture content (Table 1). The SV decreased to 2.15, when particle size of cotton stalks decreased to 1 mm with 75% moisture content, due to the high cellulose loss with respect to lignin degradation. SV was 2.13 when particle size increased to 10 mm, insignificantly different from 1 mm. Increase in moisture content decreased the SV, and this may be due to reduction in lignin degradation compared to cellulose loss caused by decrease in oxygen diffusion and inhibition of lignolytic enzymes.

#### Effect of supplements

Effect of supplements, such as Cu^2+^, Mn^2+^, gallic acid (GA), and veratryl alcohol (VA), with varying concentration 0.1 mM to 2 mM/g cotton stalks (solid) on degradation of cotton stalks was studied (Table 2). Cu^2+^ at 0.5 mM/g with lignin degradation 33.74 ± 1.17% (w/w), SV of 3.15; gallic acid (GA) at 0.5 mM/g with lignin degradation 31.20 ± 0.24% (w/w), SV of 2.76; and veratryl alcohol at 1 mM/g with lignin degradation 32.24 ± 3.22% (w/w), SV of 2.61 were optimal after 20 days. Mn^2+^ supplements had not any effect on lignin degradation. Results showed that the addition of Cu^2+^, gallic acid, and veratryl alcohol enhanced the lignin degradation during pretreatment, but the SV was higher in the case of Cu^2+^ due to low cellulose degradation. The addition of higher concentration of supplements decreased the lignin degradation which could be due to the inhibition of fungal growth and lignolytic enzyme activity.

**Table 2:** Effect of supplements on degradation of cotton stalks by *D. flavida* having 5 mm particle size, 75% moisture content, and 20 days pretreatment, 28 °C

Supplements (concentration)	Degradation (%)^a^				S.V^b^
	Dry mass	Cellulose	Hemicellulose	Lignin	
Cu^2+^ (0.0 mM/g)	14.60 ± 1.00	11.70 ± 1.30	22.92 ± 1.65	29.88 ± 0.97	2.55
Cu^2+^ (0.1 mM/g)	15.13 ± 1.03	12.99 ± 0.52	27.31 ± 1.55	29.21 ± 1.13	2.24
Cu^2+^ (0.5 mM/g)	15.86 ± 1.80	10.70 ± 1.93	25.22 ± 2.32	33.74 ± 1.17	3.15
Cu^2+^ (1.0 mM/g)	14.80 ± 0.80	10.74 ± 0.92	22.46 ± 1.22	27.21 ± 0.71	2.53
Cu^2+^ (2.0 mM/g)	12.80 ± 0.52	10.49 ± 0.12	21.91 ± 0.71	25.61 ± 1.39	2.44
GA (0.0 mM/g)	14.60 ± 1.00	11.70 ± 1.30	22.92 ± 1.65	29.88 ± 0.97	2.55
GA (0.1 mM/g)	14.86 ± 3.63	13.04 ± 0.29	27.07 ± 2.23	31.56 ± 1.66	2.42
GA (0.5 mM/g)	15.13 ± 0.98	11.29 ± 0.84	22.05 ± 0.62	31.20 ± 0.24	2.76
GA (1.0 mM/g)	14.13 ± 1.20	12.24 ± 0.86	26.45 ± 1.87	28.23 ± 1.18	2.30
GA (2.0 mM/g)	10.46 ± 0.90	9.43 ± 1.02	20.07 ± 1.31	23.18 ± 1.74	2.45
Mn^2+^ (0.0 mM/g)	14.60 ± 1.00	11.70 ± 1.30	22.92 ± 1.65	29.88 ± 0.97	2.55
Mn^2+^ (0.1 mM/g)	14.93 ± 1.36	12.02 ± 1.31	23.79 ± 1.16	28.30 ± 1.61	2.35
Mn^2+^ (0.5 mM/g)	14.20 ± 1.31	11.81 ± 1.22	21.17 ± 2.44	27.05 ± 0.89	2.29
Mn^2+^ (1.0 mM/g)	12.73 ± 0.30	10.87 ± 0.58	18.09 ± 0.53	24.20 ± 1.51	2.22
Mn^2+^ (2.0 mM/g)	9.53 ± 0.50	8.94 ± 0.58	14.40 ± 0.95	18.23 ± 0.94	2.03
VA (0.0 mM/g)	14.60 ± 1.00	11.70 ± 1.30	22.92 ± 1.65	29.88 ± 0.97	2.55
VA (0.1 mM/g)	14.73 ± 1.70	12.42 ± 1.06	26.96 ± 2.14	28.21 ± 0.88	2.27
VA (0.5 mM/g)	15.06 ± 0.10	13.13 ± 0.65	26.27 ± 2.60	31.12 ± 2.10	2.37
VA (1.0 mM/g)	15.26 ± 1.02	12.34 ± 0.91	27.43 ± 0.93	32.24 ± 3.22	2.61
VA (2.0 mM/g)	16.13 ± 0.23	11.71 ± 0.31	28.17 ± 1.00	29.54 ± 1.73	2.52

^a^Data are mean values ± standard error of three replicates

^b^Selectivity value

### Lignocellulolytic enzyme production

The effect of particle size, moisture content, and supplements on lignocellulolytic enzyme production during pretreatment of cotton stalks biomass was studied.

#### Effect of particle size and moisture content

**Fig. 1:**
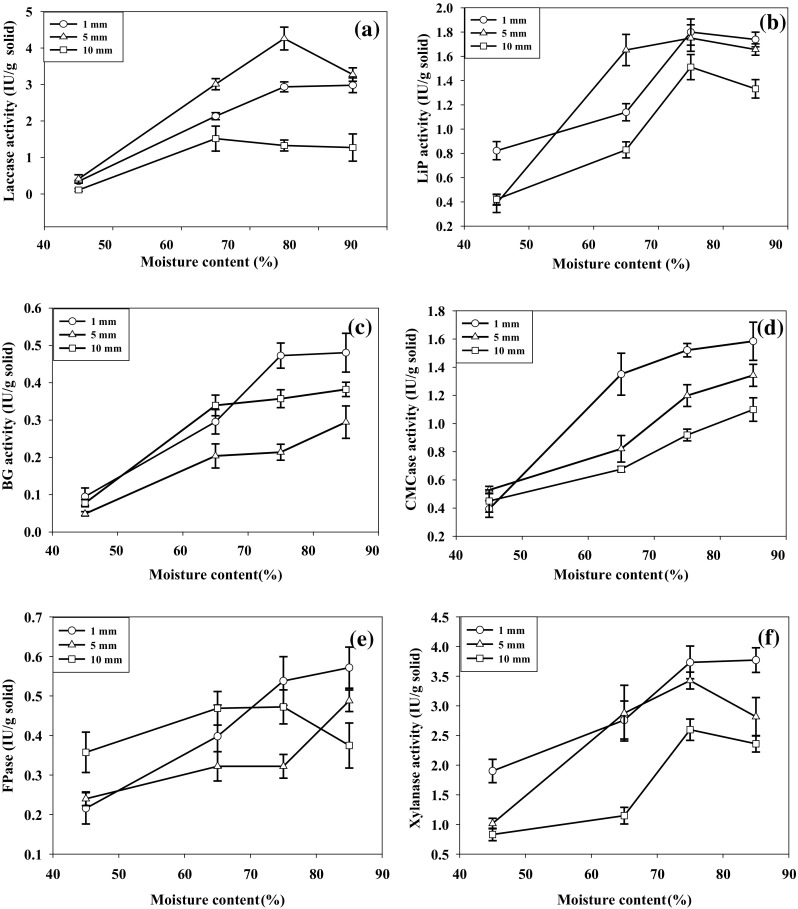
Effect of particle size and moisture content on peak activity of lignocellulolytic enzyme activities during pretreatment with *D. flavida* (DF-2) at 28 °C. **a** Laccase, **b** lignin peroxidase (LiP), **c** β-glucosidase (BG), **d** carboxymethyl cellulase (CMCase), **e** filter paper hydrolase (FPase), **f** xylanase

Laccase and lignin peroxidase (LiP) activities were detected during pretreatment of cotton stalks, whereas manganese peroxidase (MnP) activity was undetected. The highest laccase activity 4.26 ± 0.38 IU/g solid after 10 days (Fig. 1a), LiP activity 1.75 ± 0.11 IU/g solid after 15 days (Fig. 1b), and lignin degradation 29.88 ± 0.97% (w/w) after 20 days (Table 1) were observed in cotton stalks having particle size 5 mm with 75% moisture content. Laccase activity decreased in 1 and 10 mm particles. Small increase in LiP activity was observed in 1 mm particles at 75% moisture content resulted insignificant increase in lignin degradation 30.47 ± 3.31% (w/w). During first 10 days of pretreatment, although significant lignolytic enzyme activities were detected, but lignin degradation was slow in all fungal cultures. This might be due to large molecular weight lignolytic enzymes were not able to enter into the undecayed cotton stalks (Arora et al. 2002). The initial slow depolymerization of lignin was might be due to the reactive oxygen species (ROS) produced by fungi as well as free radicals (peroxyl, hydroxyl, etc.) produced by peroxidase (LiP) enzyme (Hammel et al. 2002). Due to the action of ROS and free radicals, the porosity of cotton stalks increased allowing the other higher molecular size lignolytic enzymes, such as laccase to penetrate into the cell wall of cotton stalks. In the case of production of laccase using olive leaves as substrate, the highest laccase activity was reported in olive leaves having particle size ranging 3.4–4.4 mm (Aydınoğlu and Sargin 2013). The optimal particle size and moisture content for the highest lignolytic enzyme activity in this study were 5 mm and 75%, respectively.

The β-glucosidase (BG), carboxymethyl cellulase (CMCase), and filter paper hydrolase (FPase) activities increased with decrease in the particle size (Fig. 1). The highest BG, CMCase, and FPase activities 0.48 ± 0.05, 1.58 ± 0.13, and 0.57 ± 0.05 IU/g solid, respectively (Fig. 1c–e) with the highest cellulose loss 15.88 ± 4.00% (w/w) (Table 1) were observed in particle size 1 mm at 85% moisture content after 20 days. Xylanase activity was also higher in smaller particles at high moisture content. The highest xylanase activity 3.77 ± 0.21 IU/g solid after 10 days (Fig. 1f) with hemicellulose loss of 30.08 ± 1.31% (w/w) after 20 days observed in particle size 1 mm at 85% moisture content. The cellulolytic activity was lower in 5 and 10 mm particles at 75% moisture content resulting decreased cellulose loss [11.70 ± 1.30 and 9.91 ± 1.09% (w/w), respectively]. The increase in cellulolytic activity with decrease in particle size might be due to increased surface area of lignocellulosic biomass exposed cellulose for fungal consumption.

#### Effect of supplements

Laccase activity increased when GA and Cu^2+^ were supplemented to cotton stalks, whereas small increase in lignin peroxidase (LiP) activity observed when veratryl alcohol (VA) was supplemented. MnP activity was not induced with the addition of Mn^2+^.

**Fig. 2:**
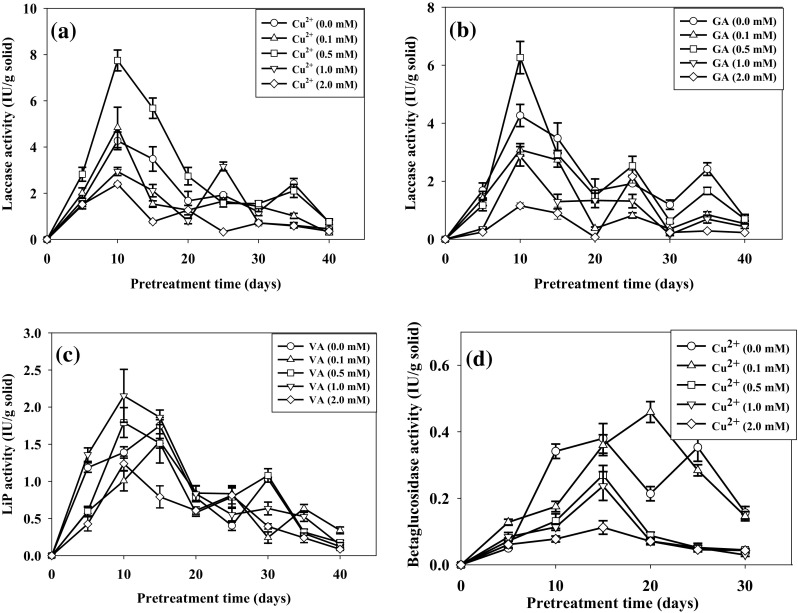

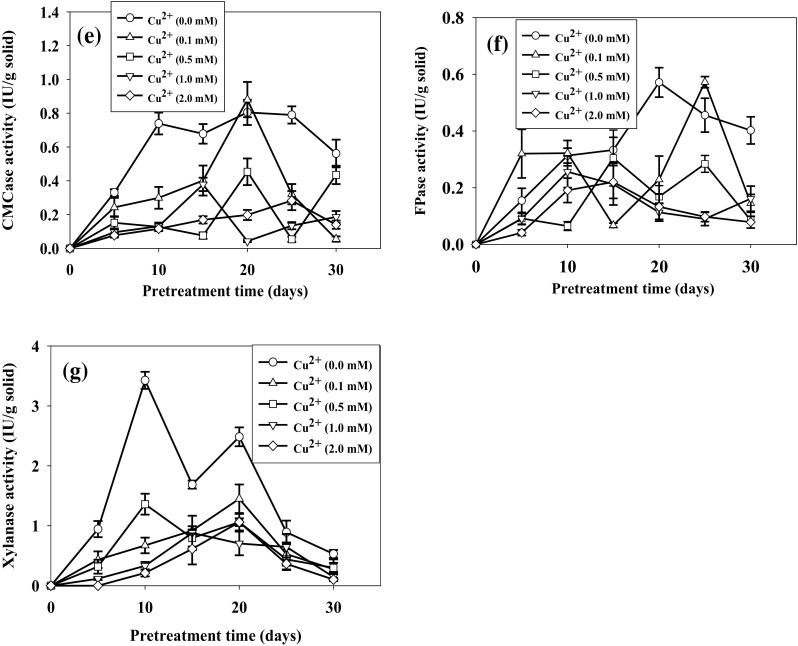
Effect of supplements on lignocellulolytic enzyme activities during pretreatment with *D. flavida* (DF-2) (particle size of 5 mm and moisture content of 75%, at 28 °C). **a**, **b** Laccase, **c** lignin peroxidase (LiP), **d** β-glucosidase (BG), **e** carboxymethyl cellulase (CMCase), **f** filter paper hydrolase (FPase), and **g** xylanase

Cu^2+^ (0.5 mM/g) and GA (0.5 mM/g) were responsible for the highest laccase activity of 7.74 ± 0.45 and 6.26 ± 0.55 IU/g solid, respectively, after 10 days of pretreatment (Fig. 2a, b) corresponded to the highest lignin degradation 33.74 ± 1.17% (w/w) and 31.20 ± 0.24% (w/w), respectively, after 20 days. The increase in laccase activity might be due to transcriptional regulation of laccase gene in the presence of Cu^2+^ (Piscitelli et al. 2011). The continuous stability of laccase activity was also observed with Cu^2+^ supplement. This could be due to the inhibition of extracellular proteolytic enzymes in the presence of Cu^2+^ that resulted inhibition of laccase degradation (Palmieri et al. 2001). Cu^2+^ has been reported for higher laccase production by *D. flavida* in rice bran media at 0.32 mM (Singha and Panda 2013), by *Trametes versicolor* at 0.4 mM (Collins and Dobson 1997), and by *Pleurotus ostreatus* at 1 mM (Baldrian and Gabriel 2002). Gallic acid at 0.1 mM induced more than threefolds increase in laccase activity by *Trametes velutina* (Yang et al. 2013). The highest LiP activity during pretreatment was 2.15 ± 0.35 IU/g solid at 1 mM/g VA (Fig. 2c) with 32.24 ± 3.22% (w/w) lignin degradation, not significantly different from without supplementation. It has been reported that VA was not an inducer for LiP (Cancel et al. 1993). Slight increase in LiP activity observed could be due to the stabilization of LiP by VA (Haemmerli et al. 1986). In addition to increased laccase activity, Cu^2+^ inhibited the cellulolytic activity (Fig. 2d–g). BG, CMCase, FPase, and xylanase activities decreased to 0.08 ± 0.01, 0.45 ± 0.07, 0.17 ± 0.04, and 1.06 ± 0.13 IU/g solid after 20 days with supplement of 0.5 mM Cu^2+^, resulting decreased cellulose loss to 10.70 ± 1.93%. Cellulolytic activity significantly decreased when the concentration of Cu^2+^ increased to 2 mM/g. VA and GA have lesser effect on cellulolytic activity. The decrease in cellulolytic activity with the addition of Cu^2+^ could be due to the decreased pH of the cotton stalks during pretreatment which inhibited the activity of cellulases (Geiger et al. 1998). The overexpression of laccase and LiP in DF-2 in the presence of supplements and inhibition of cellulases was responsible for the higher delignification and increased SV.

### Enzymatic saccharification

**Fig. 3:**
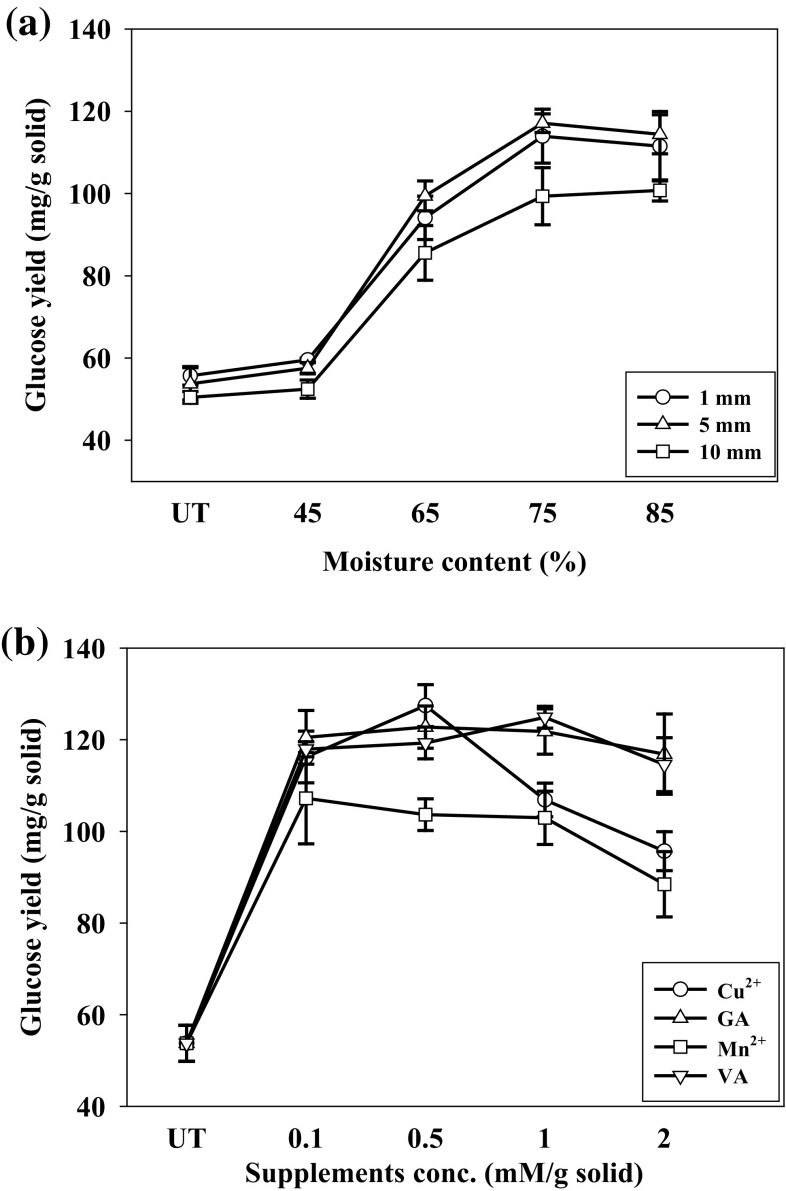
Effect of particle size, moisture content, and supplements on enzymatic hydrolysis of cotton stalks pretreated with *D. flavida* (DF-2) for 20 days

Cotton stalk particles 1, 5, and 10 mm pretreated for 20 days at 75% moisture content were enzymatically hydrolyzed. The glucose yield 117.09 ± 2.26 mg/g pretreated stalks were obtained from particle size 5 mm (Fig. 3a) corresponded to lignin degradation 29.88 ± 0.97% (w/w). The glucose yield of 113.94 ± 6.55 and 99.34 ± 6.95 mg/g was obtained from stalks having particles 1 and 10 mm with lignin degradation 30.47 ± 3.31 and 21.16 ± 0.12% (w/w), respectively. The difference between glucose yield from 1 and 5 mm particles was insignificant, but the glucose yield from 10 mm decreased. Decrease in particle size increased the surface area and provided more accessible cellulose for enzymatic saccharification, but higher cellulose degradation also occurred due to higher production of cellulases during pretreatment. This could be the reason for lower glucose yield from 1 mm than from 5 mm particles. The glucose yield from cotton stalks with 45% moisture content was almost the same as untreated cotton stalks. The glucose yield increased when moisture content was increased. The optimum particle size and moisture content of cotton stalks for maximum glucose yield were 5 mm and 75%, respectively. No significant increase in glucose yield was observed when the pretreatment time increased to 40 days.

**Fig. 4:**
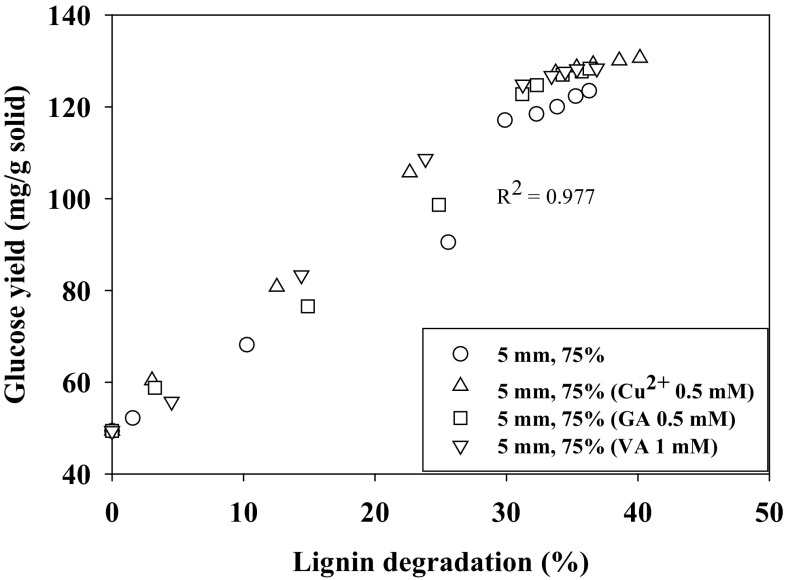
Relation between glucose yield after enzymatic hydrolysis and lignin degradation of cotton stalks (particle size 5 mm and moisture content 75%) at different conditions of pretreatment (*R* ^2^ = 0.977)

Cu^2+^ supplement 0.5 mM/g solid increased glucose yield after enzymatic saccharification to 127.44 ± 4.56 mg/g due to increased lignin degradation 33.74 ± 1.17% (w/w). Lower concentration of Cu^2+^ (0.1 mM/g) did not affect the glucose yield, but higher concentration (2 mM/g) decreased glucose yield (Fig. 3b). Gallic acid (GA) supplement 0.5 mM/g solid increased the glucose yield to 122.75 ± 4.61 mg/g due to increased lignin degradation 31.20 ± 0.24% (w/w). Small increase in glucose yield was observed when veratryl alcohol was supplemented. Cu^2+^, gallic acid, and veratryl alcohol supplements enhanced the lignolytic enzymes production/lignin degradation enabling the penetration of hydrolytic enzymes to increase the glucose yield. This was verified from a correlation between glucose yield and lignin degradation (Fig. 4). No significant increase in glucose yield was observed when the pretreatment time increased to 40 days.

### Characterization of biomass

**Fig. 5:**
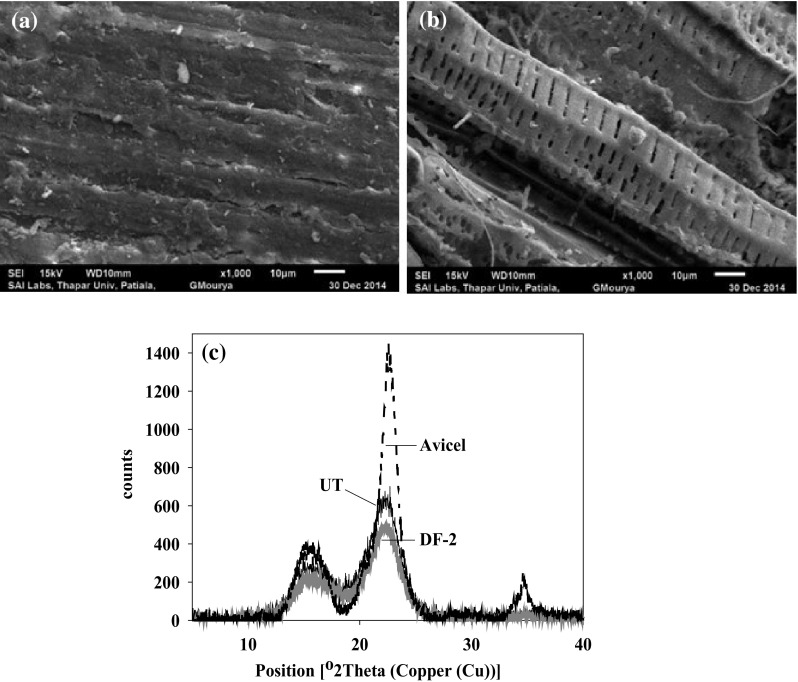
Characterization of cotton stalks. **a** SEM (untreated), **b** SEM (pretreated), and **c** XRD

The surface morphologies of cotton stalks were analyzed by SEM. Cotton stalks became porous after pretreatment (Fig. 5a, b) and accessibility of hydrolytic enzymes to the cellulose increased. Structural characterization of cellulose in untreated and pretreated cotton stalks was done on the basis of percentage crystallinity (% Cr) and crystallinity index (C.I.) (XRD analysis) taking pure micro-crystalline cellulose (Avicel) as reference (Fig. 5c). The % Cr and C.I. of avicel was 95.2% and 0.95. The % Cr and C.I. of untreated cotton stalks and cotton stalks pretreated with DF-2 were 78 and 75; 0.72 and 0.66, respectively, showed the lowering of crystallinity of cotton stalks due to degradation of lignin after pretreatment.

## Conclusion

Higher degradation of lignin with minimum cellulose loss, i.e., high SV/recovery of carbohydrate achieved using optimal particle size, moisture content, and media supplements in the pretreatment of cotton stalks by white-rot fungus *D. flavida* (DF-2). Particle size, moisture content, and media supplements influenced the lignocellulolytic enzymes for vital role in high selectivity. More than threefolds increase in glucose yield was obtained from pretreated cotton stalks after enzymatic saccharification. The problems of oxygen diffusion, metabolic heat accumulation, and reduced moisture contents during the solid-state fermentation also could influence lignocellulolytic enzymes production. Further research in this direction is required for better understanding the parameters and design of an efficient pretreatment process.

## Electronic supplementary material

Below is the link to the electronic supplementary material.

Supplementary material 1 (PDF 202 kb)

Supplementary material 2 (PDF 67 kb)

Supplementary material 3 (PDF 86 kb)
