# Supplementary material for: Effect of particle size, moisture content, and supplements on selective pretreatment of cotton stalks by Daedalea flavida and enzymatic saccharification
Source: 3 Biotech. 2016 Nov 3;6(2):235. doi: 10.1007/s13205-016-0548-x (PMC5095101; doi:10.1007/s13205-016-0548-x)
Supplement: Supplementary file 1 — Supplementary material 1 (PDF 202 kb) [file 13205_2016_548_MOESM1_ESM.pdf]

Title: Effect of particle size, moisture content and supplements on selective pretreatment of cotton stalks by *Daedalea flavida* and enzymatic saccharification

Journal name: 3 Biotech

Harmanpreet Meehnian[1], Asim K. Jana[1]*, Mithu Maiti Jana[2]

[1]Department of Biotechnology, Dr B R A National Institute of Technology Jalandhar, 144011, Punjab (India)

[2]Department of Chemistry, Dr B R A National Institute of Technology Jalandhar, 144011, Punjab (India)

Email: janaak@nitj.ac.in

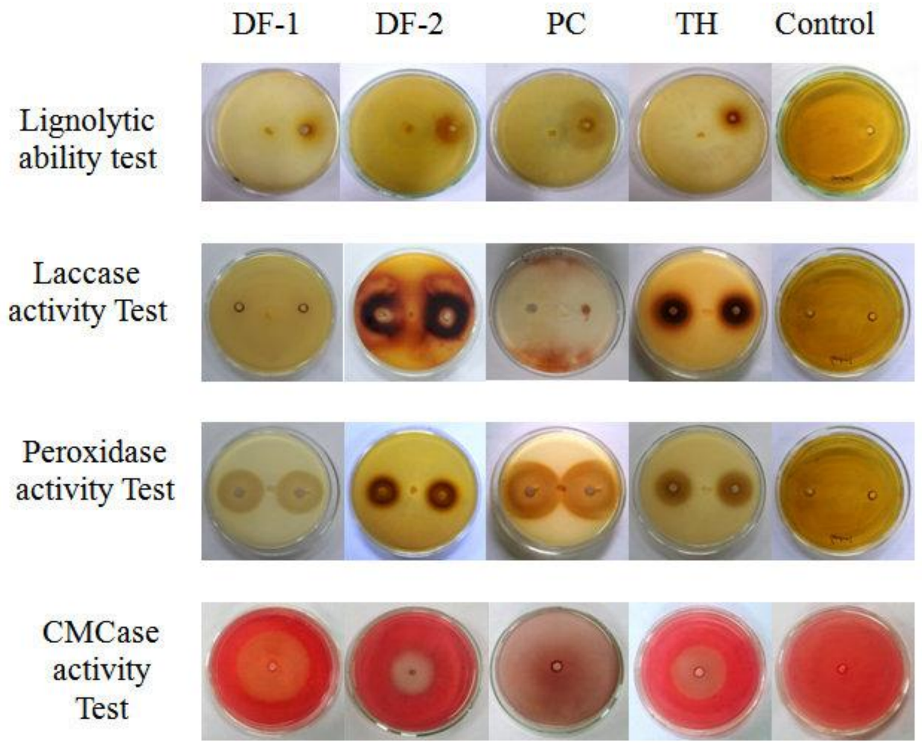

Online Resource 1 Formation of colored zones in petri plates representing ligno-cellulolytic ability of fungal strains with *Daedalea flavida* NCIM 1087 (DF-1), *Daedalea flavida* MTCC 145 (DF-2), *Phanerochaete chrysosporium* NCIM 1106 (PC), *Trametes hirsuta* MTCC 136 (TH)

The lignolytic ability, laccase activity, peroxidase activity and cellulolytic activity of fungal strains were analyzed from the diameter of characteristic colored zones produced on the full grown plates of fungal strains during tannic acid, guaiacol, pyrogallol and Congo red dye tests. After observing the diameter of zones, the strains were categorized into high, fair and low activity based upon zone diameter ≥ 35 mm, < 35 & ≥ 20 mm and < 20 mm respectively. Brown colored zones were observed around the wells containing tannic acid in full grown fungal plates which confirmed the lignolytic ability. The diameter of brown colored zone was maximum (> 35 mm) in

Phanerochaete chrysosporium (PC) plate which indicated its high lignolytic ability, whereas diameter of brown colored zone was < 35 & ≥ 20 mm in *Daedalea flavida* MTCC 145 (DF-2) and *Trametes hirsuta* (TH) showed their fair lignolytic ability. *Daedalea flavida* NCIM 1087 (DF-1) had diameter of 12 mm of brown colored zone, which showed its low lignolytic ability. Laccase activity was observed in all strains in guaiacol test except in PC. Dark purple colored zones of diameter ≥ 35 mm around the wells containing guaiacol was observed in full grown petri plate of DF-2, which showed its high laccase activity, while diameter of dark purple colored zones was 30 mm in TH, showed fair laccase activity. Dark purple colored zones of diameter 10 mm observed in full grown petri plate of DF-1 showed its low laccase activity. Golden yellow to brown colored zones were observed in all strains during pyrogallol test confirming peroxidase activity but the diameter was maximum (> 35 mm) in plate of PC, whereas diameter < 35 & ≥ 20 mm of golden yellow zones in rest of the fungal strains showed their fair peroxidase activity. Cellulolytic (CMCase) activity was observed in all strains, but yellow / halo zones of diameter > 35 mm observed along the growth in DF-1 and PC petri plates showed their high CMCase activity. Yellow / halo zone of 34 mm was observed in TH plate showed its fair CMCase activity, whereas, zone of 19 mm along the growth of DF-2 in petri plates showed its low cellulolytic activity. It was observed that different fungal strains were having different lignocellulolytic abilities. DF-2 was having high lignolytic ability with lowest cellulolytic ability among all strains.
