# Supplementary material for: Effect of particle size, moisture content, and supplements on selective pretreatment of cotton stalks by Daedalea flavida and enzymatic saccharification
Source: 3 Biotech. 2016 Nov 3;6(2):235. doi: 10.1007/s13205-016-0548-x (PMC5095101; doi:10.1007/s13205-016-0548-x)
Supplement: Supplementary file 2 — Supplementary material 2 (PDF 67 kb) [file 13205_2016_548_MOESM2_ESM.pdf]

Title: Effect of particle size, moisture content and supplements on selective pretreatment of cotton stalks by *Daedalea flavida* and enzymatic saccharification

Journal name: 3 Biotech

Harmanpreet Meehnian[1], Asim K. Jana[1]*, Mithu Maiti Jana[2]

[1]Department of Biotechnology, Dr B R A National Institute of Technology Jalandhar, 144011, Punjab (India)

[2]Department of Chemistry, Dr B R A National Institute of Technology Jalandhar, 144011, Punjab (India)

Email: janaak@nitj.ac.in

Online Resource 2 Diameter of colored zones in petri plates representing ligno-cellulolytic ability of fungal strains

Fungal strains	Tannic acid test (lignolytic ability)		Guaiacol test (laccase activity)		Pyrogallol test (peroxidase activity)		Congo-red test (CMCase activity)	
	Brown zone (dia, mm)	Activity category	Dark red / dark purple zone (dia, mm)	Activity category	Golden yellow / brown zone (dia, mm)	Activity category	Yellow / opaque zone (dia, mm)	Category
DF-1	12	L	10	L	33	F	50	H
DF-2	25	F	36	H	20	F	19	L
PC	41	H	n.d	n.d	45	H	45	H
TH	20	F	30	F	26	F	34	F

n.d : Not detected, H: High (≥ 35 mm), F: Fair (< 35 & ≥ 20 mm) and L: Low (< 20 mm)
Daedalea flavida NCIM1087 (DF1), *Daedalea flavida* MTCC 145 (DF-2), *Phanerochaete chrysosporium* NCIM 1106 (PC), *Trametes hirsuta* MTCC 136 (TH)
