# Supplementary material for: Effect of particle size, moisture content, and supplements on selective pretreatment of cotton stalks by Daedalea flavida and enzymatic saccharification
Source: 3 Biotech. 2016 Nov 3;6(2):235. doi: 10.1007/s13205-016-0548-x (PMC5095101; doi:10.1007/s13205-016-0548-x)
Supplement: Supplementary file 3 — Supplementary material 3 (PDF 86 kb) [file 13205_2016_548_MOESM3_ESM.pdf]

Title: Effect of particle size, moisture content and supplements on selective pretreatment of cotton stalks by *Daedalea flavida* and enzymatic saccharification

Journal name: 3Biotech

Harmanpreet Meehnian[1], Asim K. Jana[1]*, Mithu Maiti Jana[2]

[1]Department of Biotechnology, Dr B R A National Institute of Technology Jalandhar, 144011, Punjab (India)

[2]Department of Chemistry, Dr B R A National Institute of Technology Jalandhar, 144011, Punjab (India)

Email: janaak@nitj.ac.in

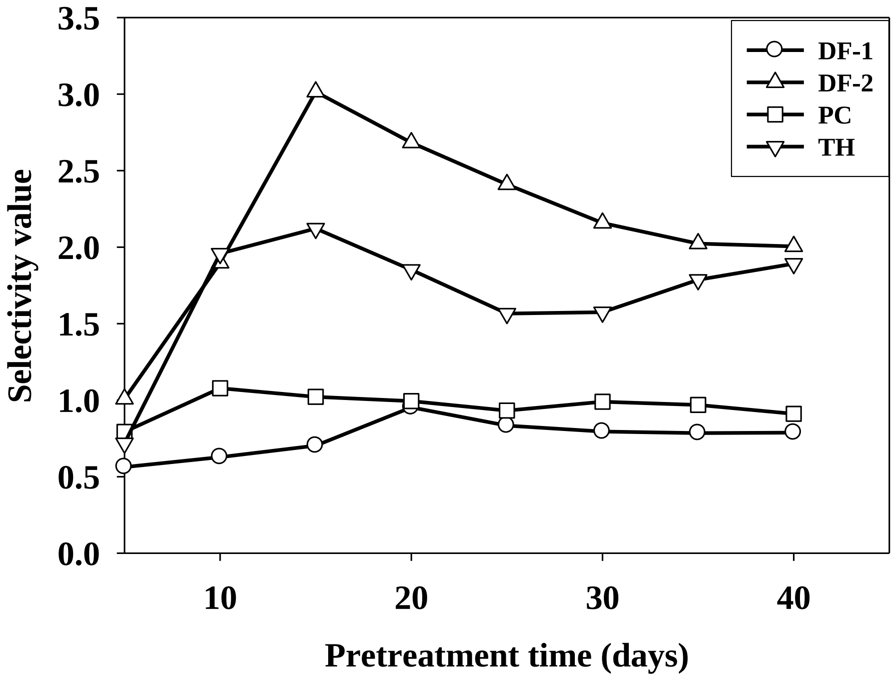

Online Resource 3 Selectivity values during pretreatment of cotton stalks with *Daedalea flavida* NCIM 1087 (DF-1), *Daedalea flavida* MTCC 145 (DF-2), *Phanerochaete chrysosporium* NCIM 1106 (PC), *Trametes hirsuta* MTCC 136 (TH)
